# *In Silico* Analysis Reveals Sequential Interactions and Protein Conformational Changes during the Binding of Chemokine CXCL-8 to Its Receptor CXCR1

**DOI:** 10.1371/journal.pone.0094178

**Published:** 2014-04-04

**Authors:** Je-Wen Liou, Fang-Tzu Chang, Yi Chung, Wen-Yi Chen, Wolfgang B. Fischer, Hao-Jen Hsu

**Affiliations:** 1 Department of Biochemistry, School of Medicine, Tzu Chi University, Hualien, Taiwan; 2 Department of Life Sciences, Tzu Chi University, Hualien, Taiwan; 3 Nanotechnology Research Center, National Dong Hwa University, Hualien, Taiwan; 4 Institute of Biophotonics, School of Biomedical Science and Engineering and Biophotonics & Molecular Imaging Research Center (BMIRC), National Yang Ming University, Taipei, Taiwan; German Institute of Human Nutrition Potsdam-Rehbruecke, Germany

## Abstract

Chemokine CXCL-8 plays a central role in human immune response by binding to and activate its cognate receptor CXCR1, a member of the G-protein coupled receptor (GPCR) family. The full-length structure of CXCR1 is modeled by combining the structures of previous NMR experiments with those from homology modeling. Molecular docking is performed to search favorable binding sites of monomeric and dimeric CXCL-8 with CXCR1 and a mutated form of it. The receptor-ligand complex is embedded into a lipid bilayer and used in multi ns molecular dynamics (MD) simulations. A multi-steps binding mode is proposed: (i) the N-loop of CXCL-8 initially binds to the N-terminal domain of receptor CXCR1 driven predominantly by electrostatic interactions; (ii) hydrophobic interactions allow the N-terminal Glu-Leu-Arg (ELR) motif of CXCL-8 to move closer to the extracellular loops of CXCR1; (iii) electrostatic interactions finally dominate the interaction between the N-terminal ELR motif of CXCL-8 and the EC-loops of CXCR1. Mutation of CXCR1 abrogates this mode of binding. The detailed binding process may help to facilitate the discovery of agonists and antagonists for rational drug design.

## Introduction

Chemokines regulate a wide range of biological and pathological processes by recruiting leukocytes to the site of injury and infection to embryogenesis, wound healing, metastasis, innate immunity and angiogenesis [1]–[3]. Chemokines carry out their function by binding to and activation of the G-protein-coupled receptors (GPCRs) on the cell surface. In humans, two types of high-affinity chemokines receptors, CXCR1 and CXCR2, belonging to the GPCR superfamily, have been identified to interact with the chemokine CXCL-8 (also known as interleukin-8, IL-8) [4]. Upon binding of CXCL-8 to the receptor on the neutrophils, CXCR1 undergoes conformational changes resulting in signal transduction by activating the Gαi subunit, leading to the release of Gβγ subunits [5]. Furthermore, CXCL-8 binding to its receptors has an effect on tumor growth by promoting angiogenesis. For example, CXCR1 has been identified as a target for blocking the formation of breast cancer stem cells and malignant melanoma that drive tumor growth and metastasis [6], [7]. Since the binding of the CXCL-8 to CXCR1 are central in various biological processes and signal transduction pathways, it will be of great importance to understand the exact interactions between CXCL-8 and CXCR1 in the structural point of view.

The secondary structure of CXCL-8 consists of a helix, three antiparallel β-strands, and a C-terminal α-helix. CXCL-8 is held together by two conserved disulfide bonds between Cys7 and Cys34 and between Cys9 and Cys50 [8]. The N-loop located after Cys7 and Cys9 is approximate 10 amino acids in length, which has been considered as a crucial binding region. The C-terminal helix plays a functional role in heparin binding and receptor interaction [9], [10]. At high concentration of CXCL-8, homodimer is the preferred state of oligomerization. Previous studies show that both, monomers and dimers of CXCL-8 are able to bind to CXCR1, with a preference for the monomer [11].

Previous studies revealed that the effect of charge-charge interaction plays a crucial role in the binding of CXCL-8 with CXCR1 [12]–[15]. The ELR motif preceding the CXC sequence on the N-terminus and the N-terminal loop (N-loop) of CXCL-8 have been validated to be significantly associated with its cognate receptor CXCR1 [12], [15]. Moreover, mutagenesis studies on CXCR1 have demonstrated that charged residues near the third and fourth extracellular loops (EC loops) are crucial for association [13], [15], [16]. Based on those previous works, the proposed mechanism for binding involves two interactions: between the N-loop of CXCL-8 and the N-terminal domain residues of the receptor CXCR1 (site I) and between the N-terminus of CXCL-8 and the EC loops residues of CXCR1 (site II) [15], [17]–[20].

The mechanisms that chemokines modulate specific biological activities are essential for understanding how GPCRs transmit signals through the lipid bilayer to the cell interior [20]. Most of earlier works only focused on the CXCL-8 binding with CXCR1 by using biochemical assay to determine the respective roles of the associated domains [8], [11], [13], [17]–[23]. The structures of monomeric and dimeric CXCL-8 have been resolved by X-ray and NMR experiments [24]–[26] as well as the probable locations of CXCL-8 bound to the N-terminal domain of CXCR1. However, the short synthetic peptides of CXCR1 are not representative to delineate the entire binding regions. The three dimensional structure of the chemokine receptor CXCR1 (residues 29∼324) has been recently determined by Opella *et al.* [27] however the structure of the N-terminal region of CXCR1 is still not yet resolved, which has been identified to play a crucial role in the binding of CXCL-8. CXCL-8 prefers to exist as a dimer in solution, while the reason for increased binding affinity as a monomer, is not known. Due to the lack of experimentally derived structural information of CXCR1, interaction between CXCL-8 and receptor is still in the dark.

Molecular simulations provide access to the analysis of protein-protein interactions. In this study, a full-length structure of CXCR1 is constructed by combining the structures from NMR experiments and those from homology modeling. Molecular docking is then performed to search for the preferable binding sites of monomeric and dimeric CXCL-8 with full-length wild-type and mutated CXCR1. In the following, the complex structures are embedded into a lipid bilayer for molecular dynamics (MD) simulations to study the interactions of monomeric and dimeric CXCL-8 with CXCR1. These various control simulations on CXCL-8 and CXCR1 in the phospholipid bilayers enable us to propose a model for the interactions between CXCL-8 and CXCR1. The results obtained by this study will also have the potential for assisting the development of CXCR1 antagonists, which then can be used for rational drug design.

## Materials and Methods

### Full-length CXCR1 structure modeling

Since the N- and C-terminal domains of rhodopsin-like GPCRs are known to be highly flexible, to date, only the bovine rhodopsin structure was resolved including N- and C-terminal domain (PDB code: 1U19), whereas others, such as CXCR1 (PDB code: 2LNL), β2AR (PDB code: 2RH1), A2AR (PDB code: 3EML) and CXCR4 (PDB code: 3ODU) lacked N- or C-terminal part. Bovine rhodopsin structure was therefore used as a template for homology modeling the receptor CXCR1 or CXCR2 [14], [28]. Moreover, previous studies from Opella *et al.* [20] also showed that as CXCL-8 comes closer to bind with CXCR1, the N-terminal domain of CXCR1 is translated to the extracellular loops of CXCR1. In this research, full-length CXCR1 was constructed by combining the NMR experiments from Opella, S. J. *et al.* (PDB code: 2LNL) [27] and homology modeling results. The homologous models for the N-terminal part (residue 2∼28) and the C-terminal part (residue 325∼347) of CXCR1 were created by using the Phyre2 web server (http://www.sbg.bio.ic.ac.uk/phyre2/) [29]. The two modeled terminal parts and CXCR1_29–324_ were then merged together to form a full-length CXCR1 structure using the MOE software package (Molecular Operating Environment, http://www.chemcomp.com). The full-length CXCR1 structure (residue 2∼347) was following embedded into a fully hydrated POPC lipid bilayer (16∶0−18∶1 diester PC, 1-palmitory-2-oleoyl-sn-glycero-3-phospho-chloine) for 50 ns MD simulation to derive a relaxation of the conformation.

### Molecular Docking

The initial favorable sites for ligands (monomeric and dimeric CXCL-8) binding to the receptor CXCR1 (wild-type and mutated) were determined by using the ‘dock proteins protocol’ from Discovery Studio 3.0 (Accelrys Inc., San Diego, CA). The ZDOCK protocol was used to conduct the rigid-body docking of two protein structures as well as clustering the poses according to the ligand position using a Fast Fourier Transformation (FFT) to perform an exhaustive six-dimensional search in the translational and rotational space between the two molecules [30]. Previous studies have demonstrated that the N-terminal domain of receptor CXCR1 plays a crucial role in ligand CXCL-8 binding [15], [17]–[19], [22], [25]. A general two-sites model was also proposed that chemokine-receptor binding involves two interactions: between the N-loop of CXCL-8 and the N-terminus of CXCR1 (site I) and between the N-terminal of CXCL-8 and the extracellular loops of CXCR1 (site II). Based on these results, the molecular docking was set to block the most part of transmembrane domain and all the intracellular residues and to allow the N-terminal domain, extracellular loops and several transmembrane residues for ligand binding. Residues except for the extracellular domain of CXCR1 (residues 38∼97, 116∼174, 206∼262, and 286∼347) were blocked to reduce the computing cost and enhance the docking accuracy. The rotational search sampling grid is used as a 15° grid which samples a total of 3600 docked poses. ZDOCK searches conformational space by rotating the ligand around its geometric center with the receptor kept fixed in space. The ZRANK function, as part of the ZDOCK protocol, is used to re-rank the docked poses. The obtained complex configurations were ranked based on a scoring function of a linear-weighted sum of van der Waals energies, electrostatics and desolvation energies. Higher scores obtained from the ZDOCK program mean that, the complex structures are of better quality. Each ZDOCK run generates 3600 poses, and are clustered into a maximum of 50 groups. The RDOCK protocol can be used subsequently for further refinement of the dozens of poses with higher ZDOCK scores, using a CHARMm-based energy minimization scheme for the optimization of intermolecular interactions. Scoring is based on a CHARMm electrostatic energy term and a desolvation energy term [31]. The RDOCK scores are defined as the summation of the electrostatic energy from the predicted complex after minimization and the desolvation energy from the complex. The structure with the lowest RDOCK scores is selected for further MD simulations. In this research three docking simulations were performed to generate the most preferable binding complex configurations, including monomer

CXCL-8 with CXCR1,
dimeric CXCL-8 with CXCR1, and
monomeric CXCL-8 with mutated CXCR1 (R199A, R203A, and D265A of CXCR1, CXCR1_mut).

### Molecular Dynamics (MD) simulations

All structures were inserted into a POPC lipid bilayer system (2×144 lipids) by removing overlapping lipids and waters molecules. For the system of monomeric CXCL-8 (PDB code: 3IL8) in complex with CXCR1, after energy minimization, 61 Na^+^ and 75 Cl^−^ ions (generating a 100 mM NaCl solution), were added to neutralize the whole system. Finally, the patches consisted of the complex structure (4305 atoms), 238 POPC lipids, and 20987 SPC-water molecules including 61 Na^+^ and 75 Cl^−^ ions (79778 atoms in total). Three independent replicates of each system starting from different initial random numbers are performed to confirm the simulation results. All simulations are run for 300 ns. Details about the number of atoms in each of the simulations are summarized in Table 1.

**Table 1:** **Summary of all simulation systems.**

System	Ligand (PDB code)	Receptor (modeled)	Complex (atoms)	POPC (lipids)	Total atoms
1	Monomeric CXCL-8 (3IL8)	CXCR1	4305	238	79778
2	Dimeric CXCL-8 (1ILQ)	CXCR1	5052	238	77759
3	Monomer CXCL-8 (3IL8)	CXCR1_mut	4249	238	79148

Detailed simulation systems are listed herein including ligands, receptor, complex atoms, POPC lipids, and total atoms of the simulation box.

All MD simulations were carried out with GROMACS-4.5.5 using Gromos96 (ffG45a3) force field with an integration step size of 2 fs. The simulations were conducted in the *NPT* ensemble employing the velocity-rescaling thermostat at constant temperature 310 K, and 1 bar. The temperature of the complex protein, lipids and the solvent were separately coupled with a coupling time of 0.1 ps. Semi-isotropic pressure coupling was applied with a coupling time of 0.1 ps and a compressibility of 4.5×10^−5^ bar^−1^ for the xy-plane as well as for the z-direction. Long-range electrostatics is calculated using the particle-mesh Ewald (PME) summation algorithm with grid dimensions of 0.12 nm and interpolation order 4. Lennard-Jones and short-range Coulomb interactions were cut off at 1.4 and 1.0 nm, respectively. The following equilibration protocol was used: (i) the temperature was gradually increased from 100 K to 200 K and 310 K. The system was run for 500 ps for the each temperature. During these simulations the complex structure remained fully restraint (k = 1000 kJ mol^−1^ nm^−2^). (ii) At 310 K the restraints kept on the complex structure via the force constant k, were released in 3 steps from k = 500 kJ mol^−1^nm^−2^ to k = 250 kJ mol^−1^nm^−2^, and finally k = 100 kJ mol^−1^nm^−2^. Each step was run for 2.0 ns. The MD simulation protocol was as followed, after energy minimization and equilibration, 300 ns production runs were carried out without any constraint on the complex structure.

The root-mean-square deviation (RMSD) of the backbone and the root-mean-square fluctuation (RMSF) of C_α_ atoms were calculated. Orientation angle of the ligand binding to the receptor was also measured to identify the orientation changes during the MD simulations. Distances between the charged groups of acidic and basic residues forming ionic interactions were measured to represent electrostatics.

The orientation angle of ligand binding to the receptor CXCR1 is defined as the angle between the unit vector along the dipole of ligand and the unit vector normal to the membrane referred to previous studies [32]. The dipole is defined as 
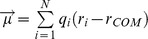
, where *q_i_* is the partial charge of each atom and *r_COM_* is the position of the center of mass of the protein.

### Molecular surface mapping

Two kinds of molecular surfaces, such as electrostatic potential map and lipophilicity map, of the complex structure snapshotted at the initial and final simulation times were generated under MOE software package platform to study the ligand-receptor interaction.

## Results

### Construction and equilibration of CXCR1 in POPC lipid bilayers

**Figure 1:**
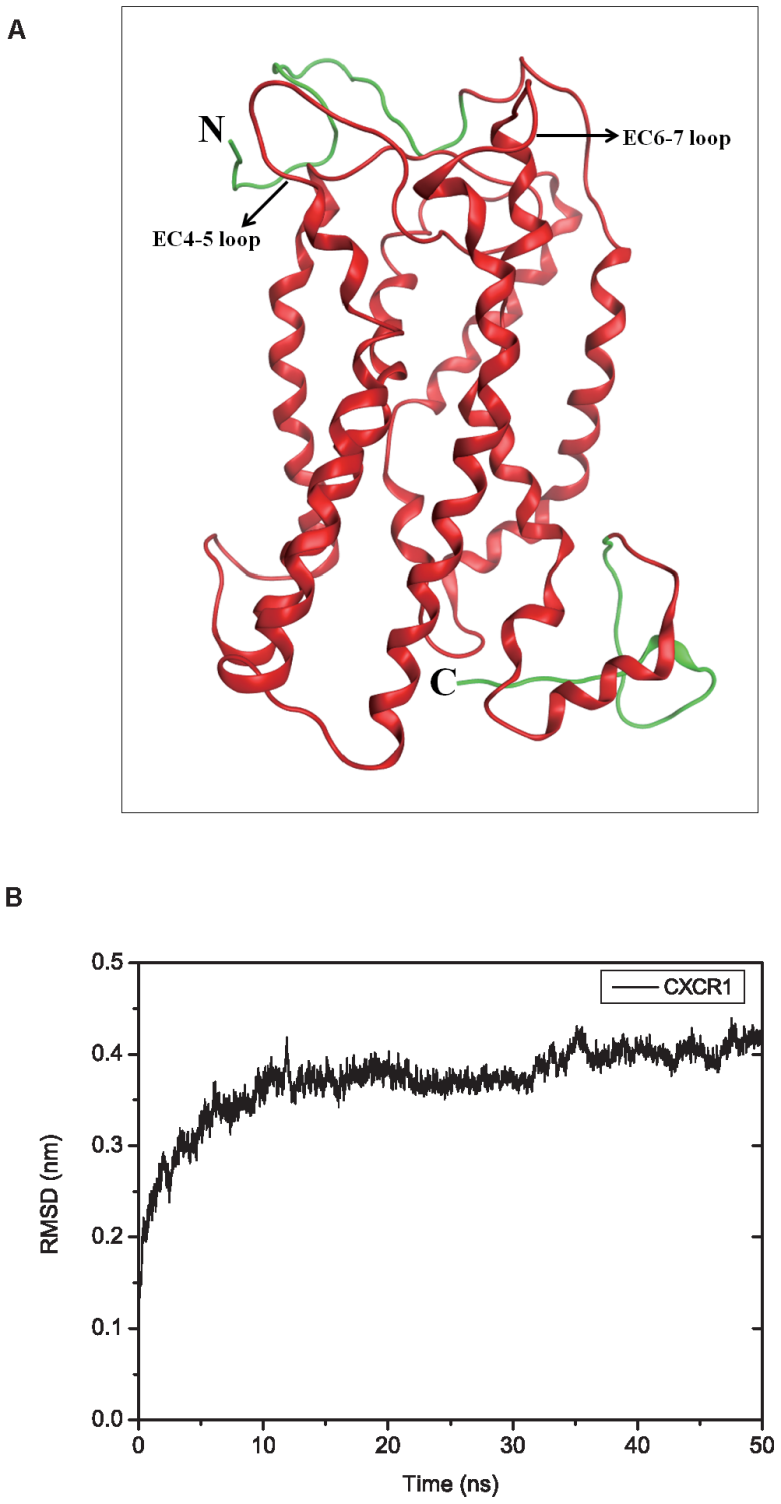
Modeled full-length CXCR1 structure and RMSD values during MD simulations. (A) Ribbon representations of the modeled full-length receptor CXCR1 (residues 2∼347) after embedded into a POPC lipid bilayer for 50 ns MD simulations. The CXCR1 is composed of the structure from the NMR experiment (residues 29∼324, red color), the N-terminal (residues 2∼28) and C-terminal (residues 325∼347) domains from homology modeling results (green color). (B) Plot of the RMSD for the backbone atoms of CXCR1 embedded into POPC lipid bilayers throughout 50 ns MD trajectory.

Sequence comparison using the Phyre2 web server show that the sequences of the N-terminal part (residue 2∼28) and the C-terminal part (residue 325∼347) of human receptor CXCR1 and bovine rhodopsin (PDB: 1U19) are 18.7% identical and 41.8% similar. Figure 1A shows the results of the modeled full-length receptor CXCR1 composed of the structure from the NMR experiment (residues 29∼324) [27], the N-terminal (residues 2∼28) and C-terminal (residues 325∼347) domains from homology modeling embedded into a POPC lipid bilayer and run for 50 ns. Both, the N-terminal and C-terminal parts of CXCR1, are long loops in random coil conformations with more negatively charged residues (D6, D11, D13, D14, D24, E25, and D26) in the N-terminus whilst more positively charged residues (K328, R333, and R335) in the C-terminus. Full-length CXCR1 embedded into a hydrated POPC lipid bilayer shows stable RMSD values in fluctuations around 0.37 nm during the first 30 ns and gradually rising up to around 0.41 nm during the last 20 ns (Figure 1B). The RMSF values per-residue of the CXCR1 indicate that the N-terminus (N-ter), extracellular loops (EC2-3, EC4-5, and EC6-7), intracellular loops (IC1-2, IC3-4, and IC5-6), and C-terminus (C-ter) generally have a higher fluctuation value compare to the transmembrane (TM) regions (Figure S1). The RMSF values fluctuate between 0.39 and 0.80 nm in the N-terminal loop due to their exposure to the solvent outside the membrane.

### MD simulations of the complex structures in POPC lipid bilayers

**Figure 2:**
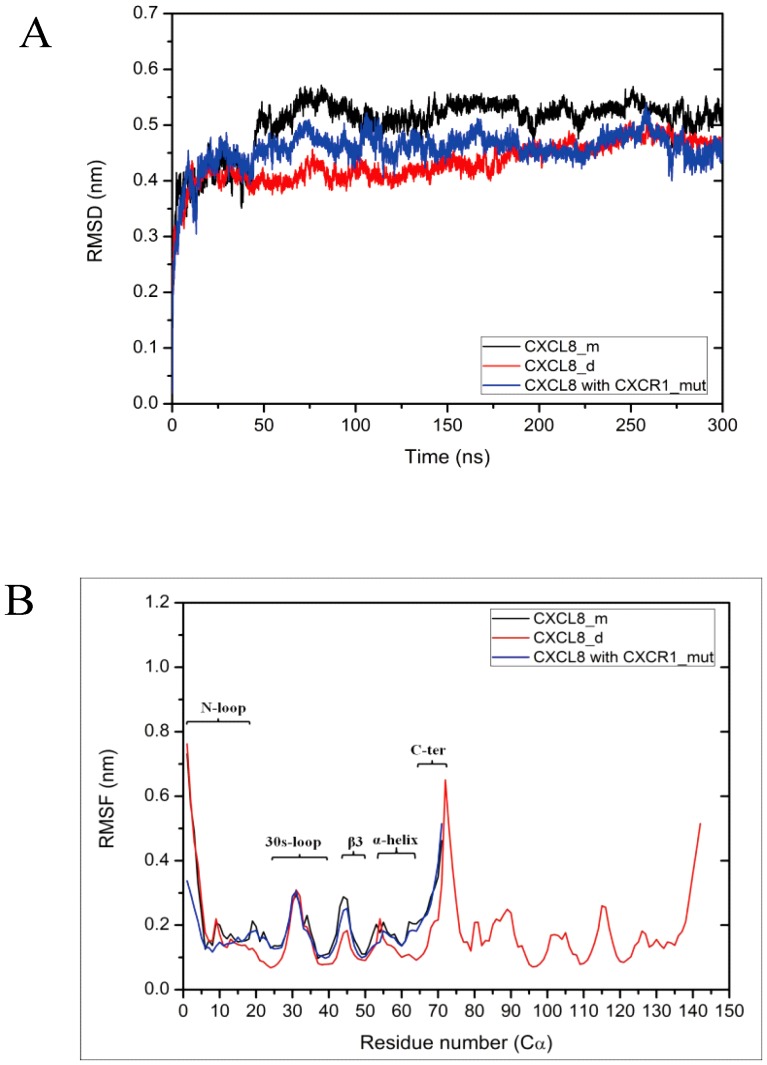
RMSD and RMSF values of CXCL-8 at various ligands binding systems during MD simulations. Calculated average RMSD of three replicates for backbone of ligands (A) as well as the average RMSF of three replicates for each residue of ligands (B) at various ligand binding receptor systems throughout the 300 ns MD simulations. Error bars of the curves are omitted for figure clarity. The locations of the N-loop, 30s-loop, β3, α-helix, and C-terminus are marked in the figure. All types of values are shown for monomeric CXCL-8 in black, dimeric CXCL-8 in red and mutated receptor CXCR1_mut (R199A, R203A, and D265A of CXCR1) in blue, respectively.

In the initial stage the rigid-body docking algorithm ZDOCK is used to generate the ligand-receptor complex structures. This docking approach generates a total of 3600 protein poses. RDOCK is used for re-ranking and refinement of the poses. Based on the ranking of RDOCK the most preferable ligand binding pose is selected for further MD simulations. The average RMSD values of three replicates for ligands at various ligand-receptor complexes fluctuate in the range of 0.41 nm to 0.53 nm when leveling off after 150 ns (Figure 2A). The average values of dimer CXCL-8 reach a plateau around 0.45 nm after 200 ns. All RMSD values of the replicates for these three systems are also shown in Figure S2A. All RMSD values for CXCR1 are leveling off after 200 ns at around 0.32 to 0.42 nm (Figure S2C).

The average RMSF values for the ligands are calculated shown in Figure 2B. The values of the N-loop (residues 1∼20) of both, the monomeric and dimeric CXCL-8 interacting with wild-type CXCR1, are higher RMSF (over 0.7 nm) than those for the protein interaction with mutated CXCR1. All detailed RMSF values of the replicates for these simulation systems are indicated in Figure S2B. Calculation of the respective RMSF values of CXCR1 shows multiple sharp maxima similar to those reported for the homology model of CXCR1 [14] (see also Figure S2D). The N- and C-terminal domains, EC-loops, as well as the IC-loops of CXCR1 fluctuate higher than 0.3 nm due to the exposure to the membrane exterior. For dimeric CXCL-8 interaction with CXCR1, the RMSF values of N-terminal domain (residues 2∼38) of CXCR1 are higher than other simulations. Furthermore, the RMSF values of C-terminal domain (residues 325∼347) of CXCR1 for the CXCR1_mut system are much higher than those for the other two simulations. A reason for this may be that CXCL-8 interacting with CXCR1_mut causes more flexible regions inside the protein.

### Orientation of ligands binding with CXCR1 receptor

**Figure 3:**
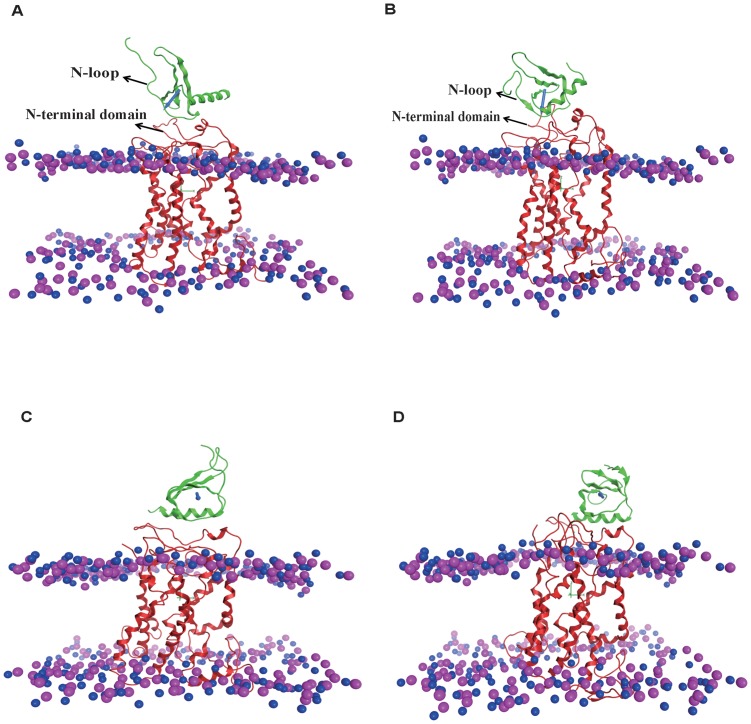
The binding orientation of ligand for various systems at different MD simulation time. Due to the similar final binding orientations of the three replicates of each system and the figure clarity, only one representative simulation run of each system is shown herein. (A) and (B): For monomeric CXCL-8 system at initial and final simulation time; (C) and (D): For mutated receptor CXCR1_mut system at initial and final simulation time. In all figures, ligands are colored with green, receptors are colored with red, and phosphorous and nitrogen atoms are colored with pink and blue, respectively. The direction of dipole moment of ligand is represented as blue arrow. The distance between the two layers is represented as the thickness of the membrane.

All the binding orientations of replicates for the various simulations at the initial and final stages are shown in Figure 3 and S3. The dipole moment is also plotted in Figure 3. The final stages of three replicates for each system are superposed to validate the results (Figure S3C∼S3E). Although the superposition of the replicates shows some differences at local loops, the whole binding orientations are quite similar. The N-loop (residue 10∼19) of monomer CXCL-8 is positioned towards the N-terminal domain of CXCR1 at the beginning of the simulation. During the simulation CXCL-8 rotates and the N-terminus of the N-loop turns towards the extracellular loops of CXCR1 (Figure 3A, 3B, and S3C). Although the orientation of dimeric CXCL-8 binding to CXCR1 is somewhat similar to that of monomeric CXCL-8, the rotation of dimeric CXCL-8 is not as much as that of monomeric CXCL-8 (Figure S3A and S3B). The three replicates also indicate similar binding orientations (Figure S3D). For CXCL-8 binding to CXCR1_mut, the initial binding position is near the N-loop and the C-terminal α-helix of CXCL-8 (Figure 3C). The binding orientation of CXCL-8 changes and moves away from the receptor CXCR1_mut during the simulation (Figure 3D and S3E). This finding implies that CXCL-8 does not prefer binding with CXCR1_mut, which is consistent with a previous study showing that the loss of binding with CXCL-8 can induce total loss of Ca^2+^ flux [13].

### The change of orientation angle of ligands during MD simulations

**Figure 4:**
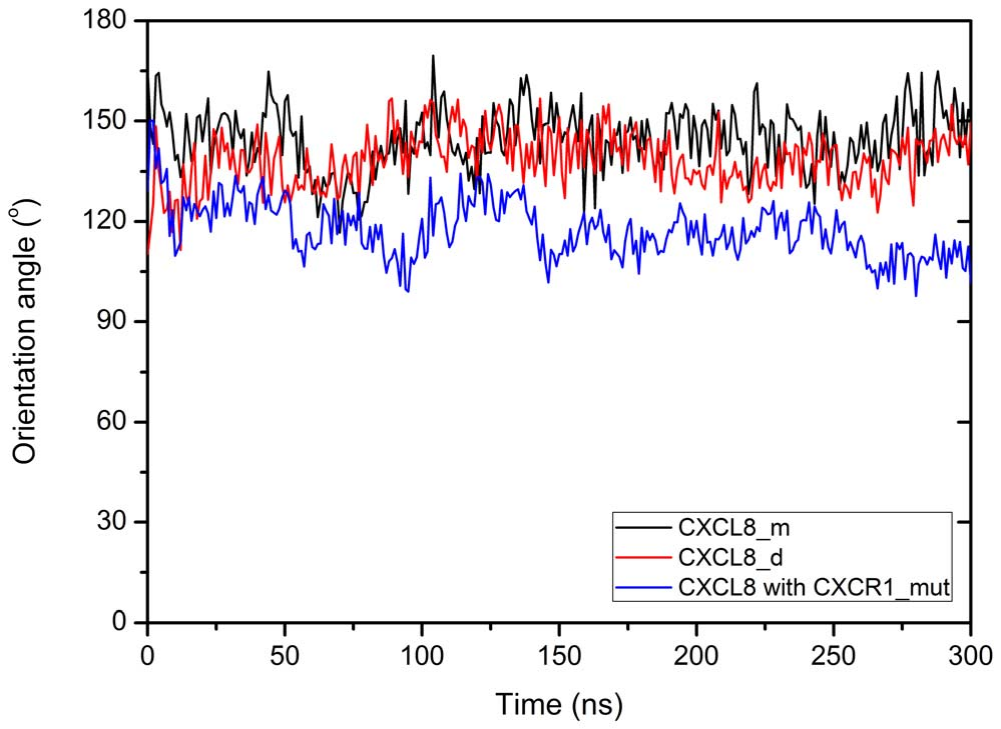
The orientation angle distribution of ligands binding with the receptor throughout the MD simulations. The orientation angle of the bound ligand is defined as the angle between the unit vector normal to the membrane and the unit vector along the dipole of ligand. The averaged curves for three replicates of each system are shown for monomeric CXCL-8 in black, dimeric CXCL-8 in red, and CXCR1_mut in blue, respectively. Error bars of the curves are omitted for figure clarity.

The orientation angle is used to quantitatively characterize the orientation of ligand binding on the receptor CXCR1. The average angle distributions used to represent the orientation change of ligand binding with the receptor CXCR1 during the MD simulations are shown in Figure 4. The changes of orientation angles with time during 300 ns are clustered every 50 ns (Table 2). For monomeric CXCL-8, the orientation angle fluctuates and gradually increases from 140.05°±7.05 (0–50 ns) to 150.44°±7.26 (250–300 ns) during the 300 ns runs. The binding orientations of monomeric CXCL-8 with CXCR1 at the beginning and at the end of the simulation are similar to those of dimeric CXCL-8. The orientation of CXCL-8 binding to mutated CXCR1 (CXCR1_mut) fluctuates and decreases from 126.48°±7.79 (0–50 ns) to 109.57°±5.32 (250–300 ns).

**Table 2:** **The changes of orientation angles with time for all simulation systems.**

System/MD time (ns)	0–50	50–100	100–150	150–200	200–250	250–300
CXCL-8 monomer	140.05°±7.09	136.49°±8.56	147.35°±7.85	145.31°±7.50	144.48°±8.08	150.44°±7.26
CXCL-8 dimer	133.58°±6.56	137.08°±7.64	144.81°±7.17	140.60°±6.95	136.09°±5.95	140.54°±7.17
CXCR1_mut	126.48°±7.79	114.66°±7.00	121.75°±6.35	115.54°±4.73	116.96°±4.74	109.57°±5.32

The changes of orientation angles are clustered every 50 ns during 300 ns MD simulations.

### Surface charge distribution of the binding complex structures

**Figure 5:**
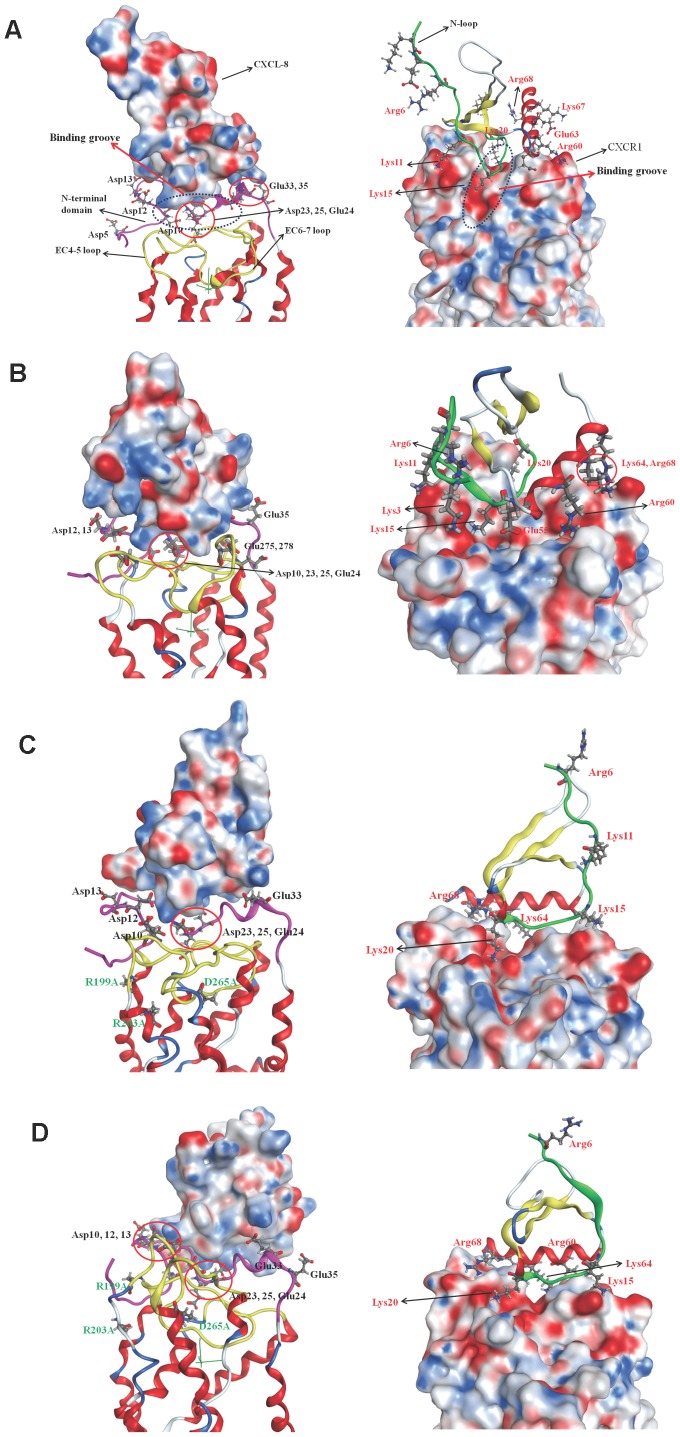
The surface charge distribution of the complex structure based on Poisson-Boltzmann equation. The complex structure is represented as ribbon structure with the N-loop of the ligand colored green, the N-terminus of receptor colored pink, and the EC-loops colored yellow. Blue color corresponds to positive and red color to negative electrostatic potential. Residues around the binding interface are labeled and shown as sticks, black is for receptor, while red is for ligand. (A): Monomeric CXCL-8 binding with CXCR1 at the initial time. Binding groove of CXCR1 is also marked. (B): Monomeric CXCL-8 binding with CXCR1 at the final simulation time. (C): CXCL-8 binding with CXCR1_mut at the initial time. (D): CXCL-8 binding with CXCR1_mut after the 300 ns runs.

Due to the similar final binding orientations of the three replicates of each system and the clarity of the simulations, only one simulation of each system is taken to analyze surface charge and lipophilicity distribution. Others are not shown herein. The surface charge distribution of the complex structure based on Poisson-Boltzmann equation for monomeric CXCL-8 binding with CXCR1 at the initial time is shown in Figure 5A. In the initial state of the binding, the end region of N-loop (residues 14∼20) of CXCL-8 binds with the groove region of N-terminal domain (residues 21∼27) of CXCR1. The C-terminal helix of CXCL-8 seems to interact with the N-terminal domain of CXCR1. Positively charged residues of CXCL-8, such as K15, K20, R60, and K64, form a positive electrostatic field near the N-loop while negatively charged residues of CXCR1, such as D11, D14, D24, D26, E25, E33, E35, form a strong negative electrostatic field around the binding groove (Figure 5A). Therefore the electrostatic interaction plays a crucial role in the initial binding of CXCL-8 with CXCR1. Extracellular loops of the CXCR1 (EC4-5 and EC6-7 loops) are more positively charged indicating these regions may prohibit from the initial binding of CXCL-8 (Figure 5A). The surface charge distribution of the complex structure after 300 ns simulation time is depicted as well in Figure 5B. Due to flipping of the extracellular loops of CXCR1 during the simulation time, the surface charge distribution of N-terminal domain of receptor CXCR1 becomes less negatively charged showing charge compensation by the ligand CXCL-8. The N-terminal residues (ELR motif) of CXCL-8 turn to approach the EC4-5 loop of CXCR1 (D194 and K197) (Figure 5B). During the 300 ns runs, the flexible N-terminal domain of CXCR1 forming a negatively charged pocket and EC-loops of CXCR1 forming a positively charged region both stabilize the CXCL-8 binding.

Due to the similar binding sites of receptor CXCR1 for dimeric CXCL-8 and monomeric CXCL-8, the surface charge distribution of dimeric CXCL-8 in complex with CXCR1 at the initial binding state is almost the same as that for the monomeric CXCL-8 (Figure S4A). The groove of N-terminal domain of CXCR1 (D24, E25, D26, and E33) is negatively charged to attract the positively charged N-loop and the C-terminal helix (K11, K15, K20, K64, and K67) of dimeric CXCL-8 for binding. After 300 ns, the rotation of the monomeric CXCL-8 is also observed in the dimeric structure. The rotation and translation of dimeric CXCL-8 allow the hydrophobic and positively charged regions to be exposed to the N-terminal domain of the receptor (Figure S4B). The ELR motif of the N-terminus of dimeric CXCL-8 also turns to approach the N-terminal domain of CXCR1, which is similar to the finding in monomeric CXCL-8.

The CXCR1_mut shows less positively charged at the EC-loops compare to that of the wild-type CXCR1 at the initial simulation time (Figure 5C). CXCL-8 does not prefer to bind to the groove of the N-terminal domain of CXCR1_mut; on the contrary, CXCL-8 binds to the lateral side of N-terminal domain of CXCR1_mut with its C-terminal helix (R60, K64, and R68). After the 300 ns, the N-terminal domain of CXCR1_mut becomes more negatively charged than that at the initial simulation time (Figure 5D). The N-terminus of CXCL-8 does not turn to approach the binding site of CXCR1_mut during the simulation time, which is validated as important for activation of the receptor [15], [19].

### Surface lipophilicity distribution of the binding complex structures

**Figure 6:**
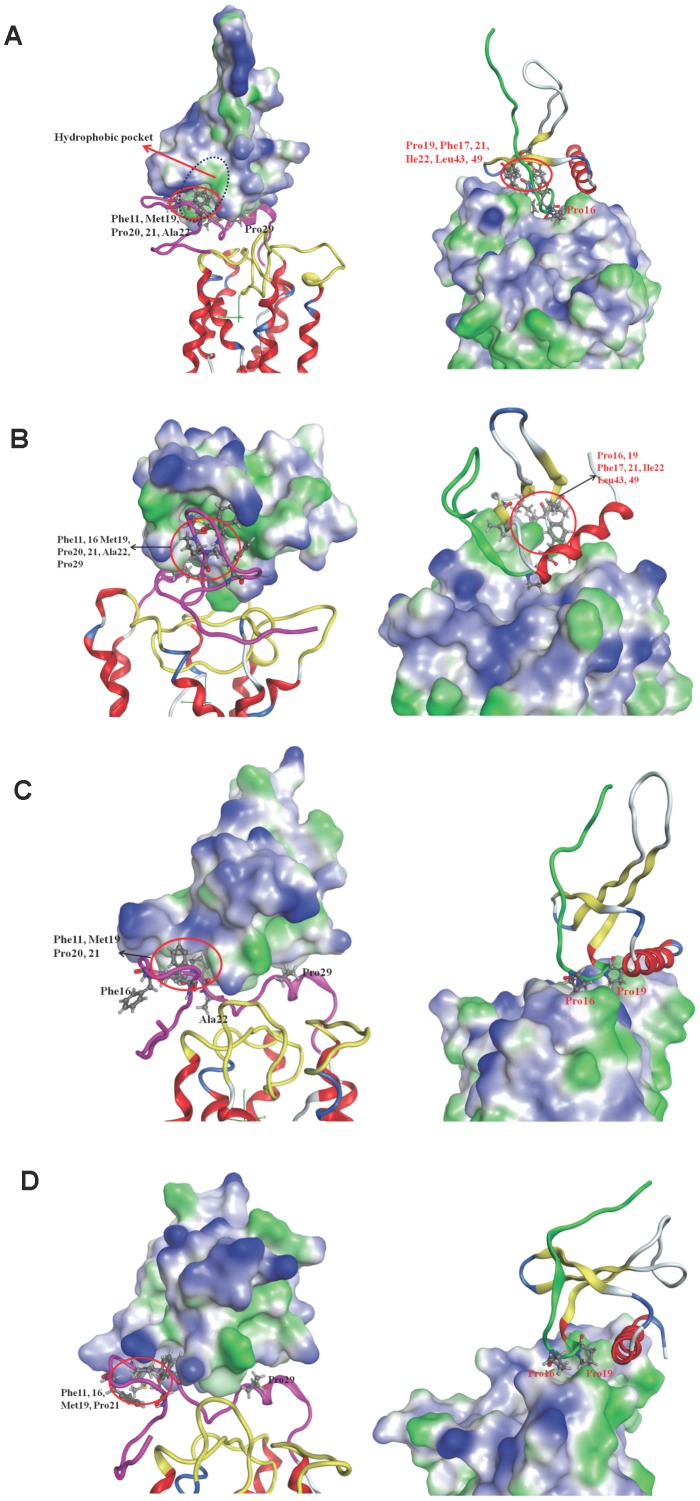
The surface lipophilicity distribution for ligand binding with receptor. The complex structure is represented as ribbon structure with the N-loop of the ligand colored green, the N-terminus of receptor colored pink, and the EC-loops colored yellow. Blue color represents the hydrophilic part while green color represents hydrophobic part. Residues around the binding interface are labeled and shown as sticks; black font is for receptor, while red font is for ligand. (A): Monomeric CXCL-8 binding with CXCR1 at the initial time. Hydrophobic pocket of ligand CXCL-8 is also marked. (B): Monomeric CXCL-8 binding with CXCR1 at the final simulation time. (C): CXCL-8 binding with CXCR1_mut at the initial time. (D): CXCL-8 binding with CXCR1_mut after the 300 ns runs.

The surface lipophilicity distribution for monomeric CXCL-8 binding with CXCR1 at the initial time is shown in Figure 6A. For the N-terminal domain of CXCR1, some hydrophobic regions, such as F16, M19, P20, P21, A22, and P29, expose outside to interact with the hydrophobic region of the N-loop of the CXCL-8 (P16, F17, P19 and F21). During the 300 ns simulations, besides the electrostatic attraction between CXCL-8 and CXCR1, CXCL-8 also rotates to form a hydrophobic pocket near the N-loop and three β strands (P16, F17, P19, F21, I22, L43 and L49) to interact with the hydrophobic part of N-terminal domain of CXCR1 (F12, F17, M20, P21, P22, A23 and P29) (Figure 6B). The hydrophobic interaction between the N-terminal domain of CXCR1 and the N-loop of CXCL-8 may play an important role in the binding process, which was mentioned in previous studies [17], [18].

The surface lipophilicity distribution for dimeric CXCL-8 binding with receptor CXCR1 at the initial time is also shown in Figure S5A. The hydrophobic contact is observed between the hydrophobic pocket of CXCL-8 and the hydrophobic region of N-terminal domain of CXCR1. After 300 ns runs, the hydrophobic contact between them still exists similar to the situation of monomeric CXCL-8 binding with receptor CXCR1 (Figure S5B).

The surface lipophilicity distributions for CXCR1_mut at the initial simulation time do not show any difference and only the mutated residues show more hydrophobic contact compared to that for wild-type CXCR1. The hydrophobic region of N-terminal domain of CXCR1_mut contacts with the hydrophilic part of CXCL-8 (between the N-loop and C-terminal helix), instead of with the hydrophobic pocket of CXCL-8 found in wild-type CXCR1 (Figure 6C and 6A), showing that this is not favorable binding. During the 300 ns MD simulations, less hydrophobic contact between them is observed suggesting that hydrophobic interaction may be not crucial in the process of CXCL-8 binding with CXCR1_mut (Figure 6D).

### Distances between the charge groups from CXCL-8 and CXCR1

**Figure 7:**
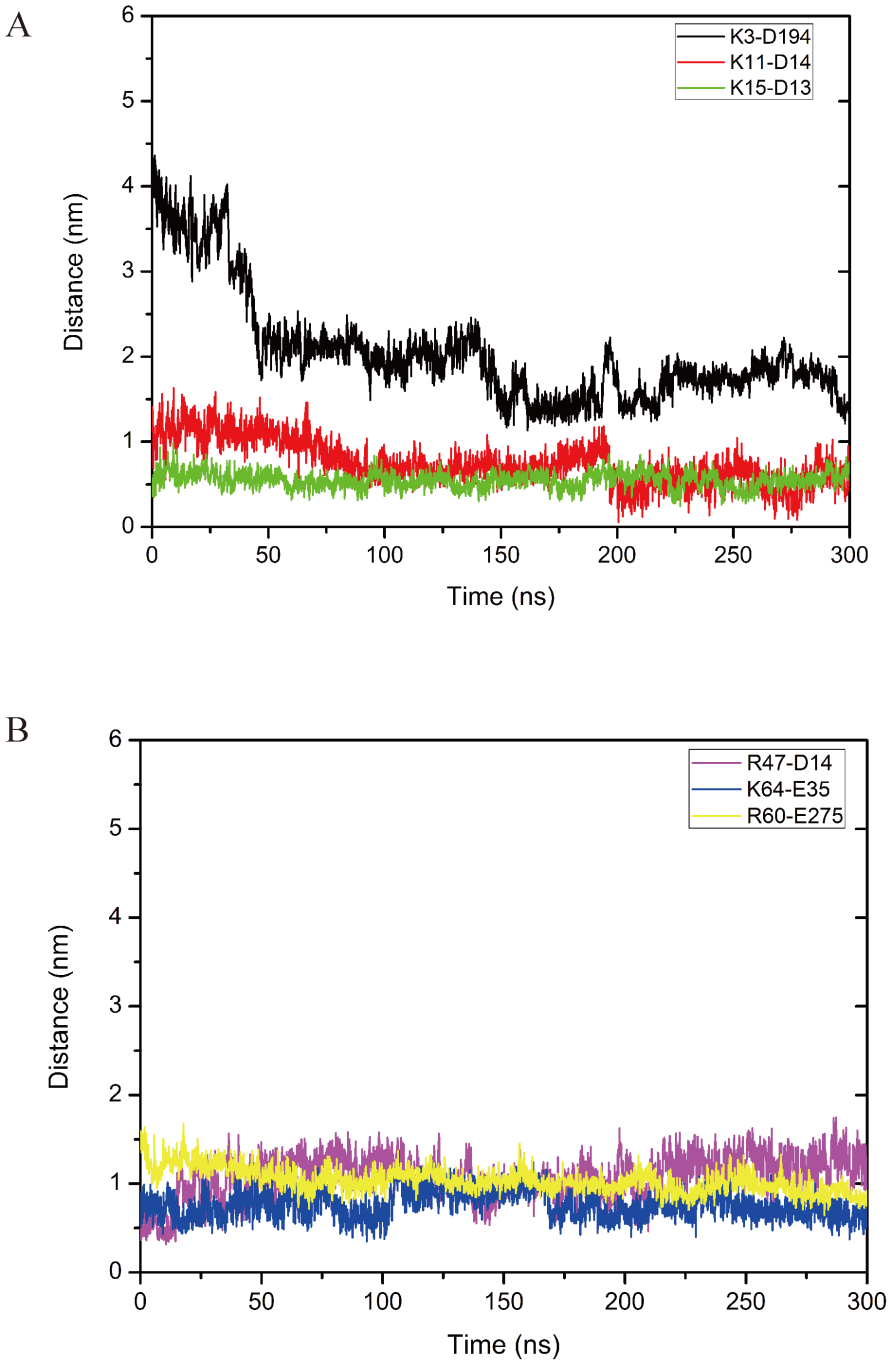
The distances between the charged groups of ligand and receptor forming electrostatic interactions. (A) Some positively charged residues of the N-loop of CXCL-8 gradually approach to the negatively charged residues of the N-terminus of CXCR1 by electrostatic interactions (K3^CXCL-8^-D194^CXCR1^ (black), K11^ CXCL-8^-D14^ CXCR1^ (red), K15^ CXCL-8^-D13^ CXCR1^ (green)) during 300 ns MD simulations. (B) Other positively charged residues of CXCL-8 interact with the negatively charged residues of CXCR1 by electrostatic interactions (R47^CXCL-8^-D14^CXCR1^ (pink), K64^ CXCL-8^-E35^ CXCR1^ (blue), R60^ CXCL-8^-E275^ CXCR1^ (yellow)) during 300 ns MD simulations. Distances are the average values with the function of time for three replicates of the system monomeric CXCL-8 binding to CXCR1. Error bars of the curves are omitted for figure clarity.

The average distances between the charged groups of selected acidic and basic residues of CXCL-8 and CXCR1 forming electrostatic interaction for the three replicates of each system are calculated with a function of time shown in Figure 7. For CXCL-8 binding to CXCR1, some positively charged residues of the N-loop of CXCL-8 (K3, R6, K11 and K15) gradually approach to the negatively charged residues of the N-terminal domain of CXCR1 (D194, D13, and D14) by electrostatic interaction (Figure 7A). The distances of K3-D194 and K11-D14 vibrate and reduce from 3.73 nm to 1.40 nm (K3-D194) and 1.41 nm to 0.49 nm (K11-D14) in the 300 ns simulation time while the distance of K15-D13 only vibrates around 0.37 nm in the simulation time due to the strong electrostatic interaction (Figure 7A). During the 300 ns simulation time, the distances for R47-D14, K64-E35, and R60-E275 also vibrate and gradually approach the binding site of CXCR1 from 0.65 nm to 0.59 nm, 0.51 nm to 0.34 nm, and 1.59 nm to 0.88 nm, respectively due to the strong electrostatic attraction shown in Figure 7B.

## Discussion

We have proposed a full-length modeled receptor CXCR1 structure (residue 2∼347) based on previously published NMR results [27] and homology modeling. A combination of docking approach and MD simulation is used to achieve a reasonable proposal for the complex structures of ligand CXCL-8 (monomer and dimer) binding to receptor CXCR1 (wild-type and mutated) embedded into a POPC lipid bilayer. The structural and dynamic properties of the ligands binding to the receptor have been further analyzed during the MD trajectories. For MD simulations, three replicates of each system with different initial velocities were performed to confirm the conformational sampling. Our previous work have verified that the MD runs changed slightly under the same simulation conditions with the different randomly initial velocities, and the RMSD seemed to develop slightly different, which is very minor to the simulations [33] and is also consistent with our results. Based on the MD simulation theory, the enough long time MD simulations are able to represent statistical significance and have been applied to various fields [34]–[36].

For monomeric CXCL-8 binding with receptor CXCR1, part of the N-loop (K15, P16, H18, K20, and F21) of CXCL-8 binds to the groove of the N-terminal domain of CXCR1 (P22, A23, D24, E25, and D26) at the initial time of MD simulation (Figure 3A). The initial binding is mainly dominated by electrostatic interactions between them (Figure 5A). The predicted initial binding site is consistent with previous experimental results [18], [20], [22], [25], [37]. After 300 ns MD runs, CXCL-8 rotates and moves closely to the binding site with ELR motif of the N-loop and 30s-loop turning to be closer to the EC4-5 and EC6-7 loops of CXCR1 (Figure 5B). In addition to electrostatic interactions, the hydrophobic pocket (P16, F17, P19, F21, L43, and L49) of the N-loop and β3 strand of CXCL-8 and other hydrophobic residues (F17, M20, P21, P22, and A23) of CXCR1 form hydrophobic contact, which may be a driving force to make CXCL-8 orientation change during the simulations (Figure 6A and 6B). Skelton *et al.* have demonstrated that P21 and P22 of CXCR1 formed hydrophobic interactions with L43 and L49 of CXCL-8 in the binding complex [25], which is consistent with our simulations. The area of the hydrophobic pocket is larger at the initial time showing that hydrophobic interaction plays an important role during the simulation time. This finding is also accordant with the previous experiments indicating that electrostatic and hydrophobic interaction mediate binding of CXCL-8 to the receptor N-terminal domain [18]. The binding site for CXCL-8 with CXCR1 seems highly conserved among different chemokines, such as CCL21-CCR7, and SDF-1α-CXCR4 [38], [39].

For dimeric CXCL-8 binding to CXCR1, part of the N-loop of dimeric CXCL-8 binding to the N-terminal domain of CXCR1 at the initial time is similar to the binding of monomeric CXCL-8. After the 300 ns runs, dimeric CXCL-8 rotates to make the ELR motif of CXCL-8 turning to be closer to the binding site of CXCR1, which is also similar to that of monomeric CXCL-8. Although previous studies showed that the binding affinity of trapped dimeric CXCL-8 to CXCR1 is lower than that of monomeric CXCL-8 [11] and binding induces dissociation of the dimer-receptor complex to the monomer-receptor complex [18], the dissociation of the dimeric CXCL-8 is unfortunately not observed during the 300 ns simulation time. Only the part the C-terminal α-helix of the CXCL-8 far away from CXCR releases the helix structure implying that dissociation of the dimer-complex to monomer-complex may need more time. Moreover, many chemokine receptors are known to form homo- or heterodimers, and these receptors provide a possibility for bound dimeric CXCL-8 to dissociate to bind with another receptor, which could not be observed in our simulation system [15].

Previous experiments demonstrated that six mutations of charged residues of CXCR1 receptor resulted in loss of binding affinity to CXCL-8 [13]. Only three of them, R199A, R203A, and D265A (CXCR1_mut), showed total loss of Ca^2+^ flux, while the remaining three, R269A, E275A, and R280A, showed reduced Ca^2+^ flux compared to wild-type CXCR1 [13]. The alanine-scanning mutagenesis of CXCR1 indicated that R199 and R203 in the third extracellular loop and D265 in the fourth loop are crucial for CXCL-8 binding and calcium mobilization. For CXCL-8 binding to CXCR1_mut, although the initial predicted preferable binding position is near the N-loop and the C-terminal α-helix of CXCL-8, which is distinct from CXCL-8 binding to wild CXCR1, the initial binding is a recognition step dominated by weak electrostatic interactions (Figure 5C). The binding orientation of CXCL-8 after the 300 ns runs is similar to that at the initial time, while after the 300 ns runs CXCL-8 departs from which at the initial time. Interestingly, it is obvious to find that the initial binding recognition is not affected significantly by the mutated residues, but the ELR motif of CXCL-8 binding to EC-loops of CXCR1 is affected by the mutations. The hydrophobic pocket of CXCL-8 does not interact with the hydrophobic region of N-terminal domain of CXCR1_mut, which is observed in the system of CXCL-8 binding to wild-type CXCR1. This suggests that hydrophobic interaction may not dominate the process for CXCL-8 binding with CXCR1_mut. The ELR motif of CXCL-8 does not turn to approach the EC-loops of CXCR1_mut, which indicates that local mutations of CXCR1 (CXCR1_mut) change the positively charged region of EC-loops to be more hydrophobic to inhibit the Ca^2+^ flux. The simulation is consistent with previous studies [13], [14].

**Figure 8:**
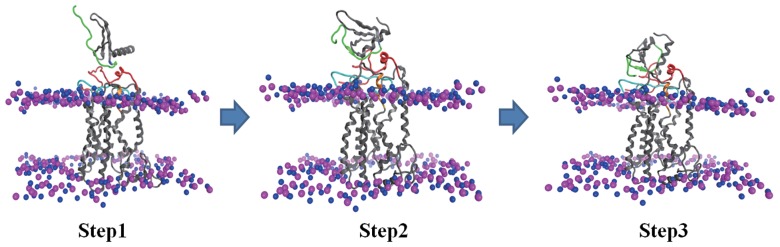
The multi-steps of mechanism of CXCL-8 binding to receptor CXCR1 in membranes. Step1: The N-loop residues of CXCL-8 bind to the N-terminus of receptor CXCR1 leading to Site-I binding. The Site-I binding is dominated mainly by the electrostatic interactions. Step2: Hydrophobic interactions near the binding site dominate to trigger the CXCL-8 rotation and conformational change. Step3: The rotation and conformational change of CXCL-8 may carry on and electrostatic interactions again dominate to attract the N-terminal ELR motif of CXCL-8 to approach the EC-loops of CXCR1 leading to Site-II binding.

Although a two-site mechanism of CXCL-8 interaction with CXCR1 has been proposed that CXCR1 binding involves two interactions: between the N-loop of CXCL-8 and N-terminal domain residues of CXCR1 (site I) and between the N-terminal of CXCL-8 and the EC-loops of CXCR1 [40], it is not fully clear how the two-site mechanism mediates affinity, selectivity, and activation of the receptor. Based on our simulations we propose the multi-steps of mechanism of CXCL-8 binding to CXCR1 (Figure 8). In the first step, the N-loop residues of CXCL-8 bind to the N-terminal domain of receptor CXCR1 leading to Site-I binding, dominated by electrostatic interactions. The Site-I binding also contributes to receptor selectivity or stabilizes a receptor signaling state in the plasma membrane. In the next step hydrophobic pocket of CXCL-8 contacts the hydrophobic region of the N-terminal domain of CXCR1. The hydrophobic interactions near the binding site dominate to trigger the CXCL-8 rotation and conformational change. In the final step, the rotation and conformational change of CXCL-8 may carry on and electrostatic interactions again dominate to attract the N-terminal ELR motif of CXCL-8 to approach the EC-loops of CXCR1 leading to Site-II binding. The Site-II binding not only potentially creates a conformational change in CXCR1 for subsequent G-protein activation, but also may trigger the C-terminal helix of CXCL-8 to bind to the sulfated cell-surface glycosaminoglycans [25], [41].

## Conclusions

In summary, computational structural modeling of biological complexes, for which experimentally derived data are rare, is still a challenging task. Modeling of a full-length membrane protein from combining the NMR experiments and homology modeling delivers a reasonable model of the receptor CXCR1 which can be used in a docking approach. Our studies suggest that in addition to electrostatic interactions, hydrophobic interactions play crucial roles in the ligand binding process. A multi-steps sequential binding mechanism is proposed, in which (i) the N-loop of CXCL-8 initially binds to the N-terminal domain of CXCR1 by electrostatic interactions, (ii) hydrophobic interactions dominate the second step resulting in the rotation and conformational change of CXCL-8, and (iii) the ELR motif of the N-terminal loop of CXCL-8 approaches the EC-loops of CXCR1 by electrostatic attractions

## Supporting Information

Figure S1 **Plot of RMSF values for the C_α_ atom of CXCR1.** The location of the terminus (N-ter, C-ter), TM helices, IC-loops, and EC-loops are marked in the figure.
(EPS)

Figure S2 **RMSD and RMSF values of CXCR1 at various ligands binding systems during MD simulations.** (A) Plot of all the RMSD of the replicates for the backbone atoms of ligands at various systems throughout the 300 ns MD simulations. (B) Plot of all the RMSF of the replicates for the C_α_ atom of CXCL-8 at various systems throughout the 300 ns MD simulations. The locations of the N-loop, 30s-loop, β3, α-helix, and C-terminus are marked in the figure. (C) Plot of all the RMSD of the replicates for the backbone atoms of CXCR1 at various ligand binding receptor systems throughout the 300 ns MD simulations. (D) Plot of all the RMSF of the replicates for the C_α_ atom of CXCR1. The location of the terminus (N-ter, C-ter), TM1, IC-loops, and EC-loops are marked in the figure. All types of values are shown for monomer CXCL-8 in black, gray, and light gray; dimer CXCL-8 in red, pink, and light red; mutated receptor CXCR1_mut (R199A, R203A, and D265A of CXCR1) in blue, cyan, and light blue respectively.
(TIF)

Figure S3 **The binding orientation of ligand for dimeric CXCL-8-CXCR1 complex system at different MD simulation time.** In the figures, dimeric CXCL-8 is colored with green, CXCR1 is colored with red, and phosphorous and nitrogen atoms are colored with pink and blue, respectively. The direction of dipole moment of ligand is represented as blue arrow. The distance between the two layers is represented as the thickness of the membrane. (A) and (B): For dimeric CXCL-8 system at initial and final simulation time; (C)∼(E): Ribbon structures of superposition of the three replicates at the final simulation time for each system. (C): monomeric CXCL-8 binding to CXCR1; (D): dimeric CXCL-8 binding to CXCR1; (E): monomeric CXCL-8 binding to mutated CXCR1. Three replicates are colored in green, blue, and yellow, respectively.
(TIF)

Figure S4 **The surface charge distributions of the complex structures.** (A) Dimeric CXCL-8 binding with CXCR1 at the initial time. (B) Dimeric CXCL-8 binding with CXCR1 after the 300 ns runs. The complex structure is represented as ribbon structure with the N-loop of the ligand colored green, the N-terminus of receptor colored pink, and the EC-loops colored yellow. Blue color corresponds to positive and red color to negative electrostatic potential. Residues around the binding interface are labeled and shown as sticks, black is for receptor, while red is for ligand.
(TIFF)

Figure S5 **The surface lipophilicity distribution for ligand binding with receptor.** (A) Dimeric CXCL-8 binding with CXCR1 at the initial time. (B) Dimeric CXCL-8 binding with CXCR1 after the 300 ns runs. The complex structure is represented as ribbon structure with the N-loop of the ligand colored green, the N-terminus of receptor colored pink, and the EC-loops colored yellow. Blue color represents the hydrophilic part while green color represents hydrophobic part. Residues around the binding interface are labeled and shown as sticks; black font is for receptor, while red font is for ligand.
(TIFF)
