# Supplementary figures and images for: In Silico Analysis Reveals Sequential Interactions and Protein Conformational Changes during the Binding of Chemokine CXCL-8 to Its Receptor CXCR1
Source: PLoS One. 2014 Apr 4;9(4):e94178. doi: 10.1371/journal.pone.0094178 (PMC3976404; doi:10.1371/journal.pone.0094178)

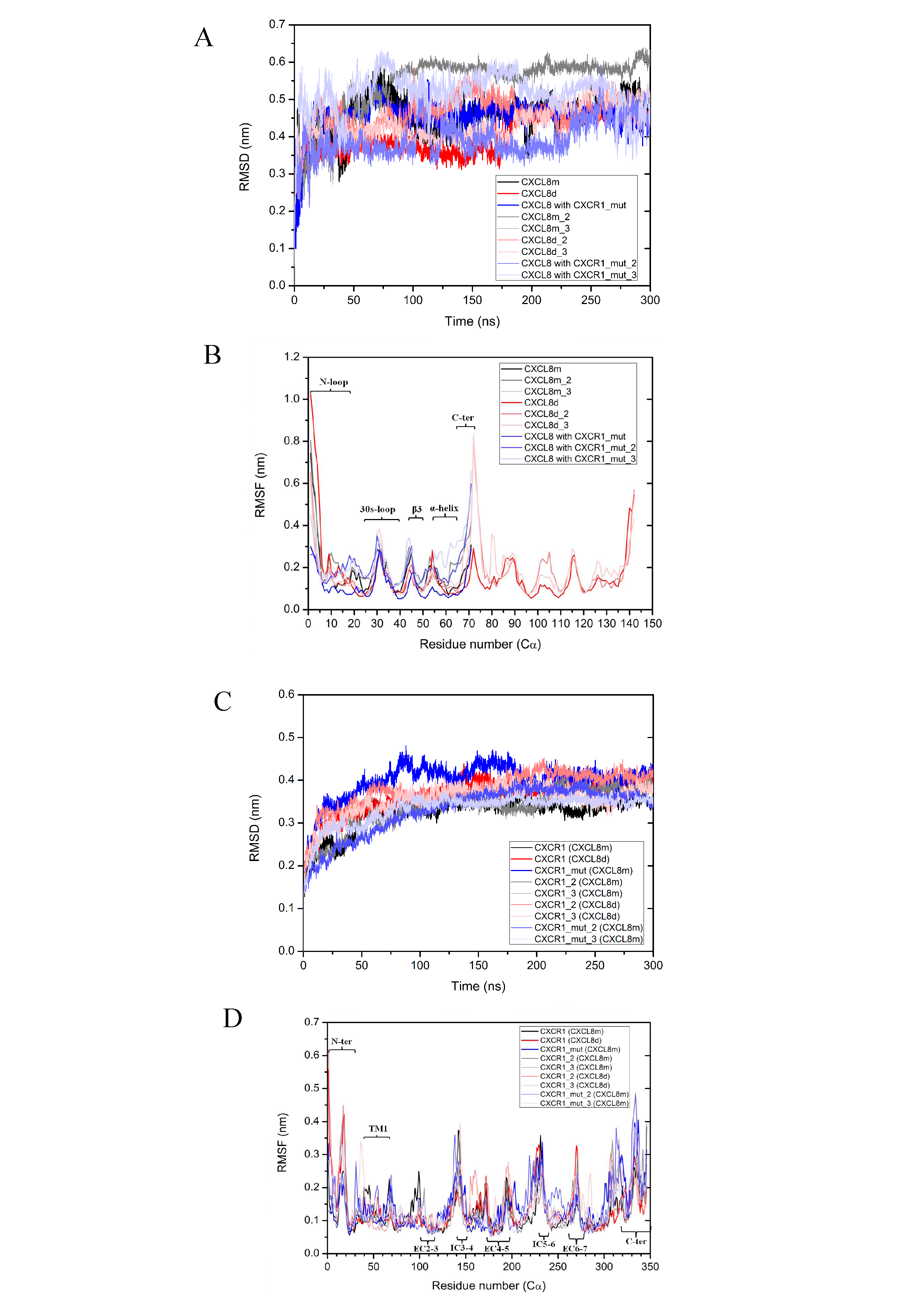

Supplement: Figure S2 — RMSD and RMSF values of CXCR1 at various ligands binding systems during MD simulations. (A) Plot of all the RMSD of the replicates for the backbone atoms of ligands at various systems throughout the 300 ns MD simulations. (B) Plot of all the RMSF of the replicates for the Cα atom of CXCL-8 at various systems throughout the 300 ns MD simulations. The locations of the N-loop, 30s-loop, β3, α-helix, and C-terminus are marked in the figure. (C) Plot of all the RMSD of the replicates for the backbone atoms of CXCR1 at various ligand binding receptor systems throughout the 300 ns MD simulations. (D) Plot of all the RMSF of the replicates for the Cα atom of CXCR1. The location of the terminus (N-ter, C-ter), TM1, IC-loops, and EC-loops are marked in the figure. All types of values are shown for monomer CXCL-8 in black, gray, and light gray; dimer CXCL-8 in red, pink, and light red; mutated receptor CXCR1_mut (R199A, R203A, and D265A of CXCR1) in blue, cyan, and light blue respectively. (TIF) [file pone.0094178.s002.tif]

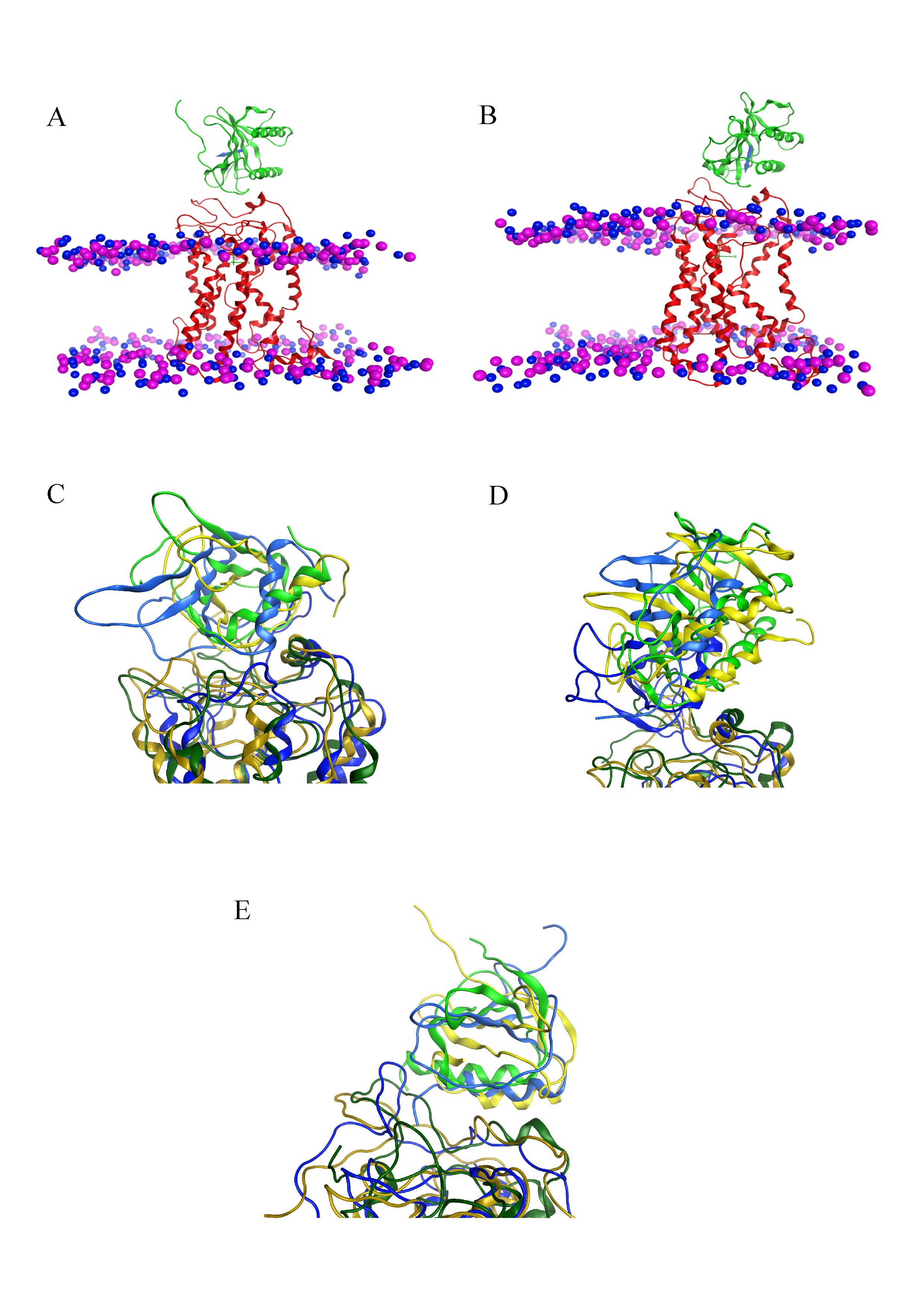

Supplement: Figure S3 — The binding orientation of ligand for dimeric CXCL-8-CXCR1 complex system at different MD simulation time. In the figures, dimeric CXCL-8 is colored with green, CXCR1 is colored with red, and phosphorous and nitrogen atoms are colored with pink and blue, respectively. The direction of dipole moment of ligand is represented as blue arrow. The distance between the two layers is represented as the thickness of the membrane. (A) and (B): For dimeric CXCL-8 system at initial and final simulation time; (C)∼(E): Ribbon structures of superposition of the three replicates at the final simulation time for each system. (C): monomeric CXCL-8 binding to CXCR1; (D): dimeric CXCL-8 binding to CXCR1; (E): monomeric CXCL-8 binding to mutated CXCR1. Three replicates are colored in green, blue, and yellow, respectively. (TIF) [file pone.0094178.s003.tif]

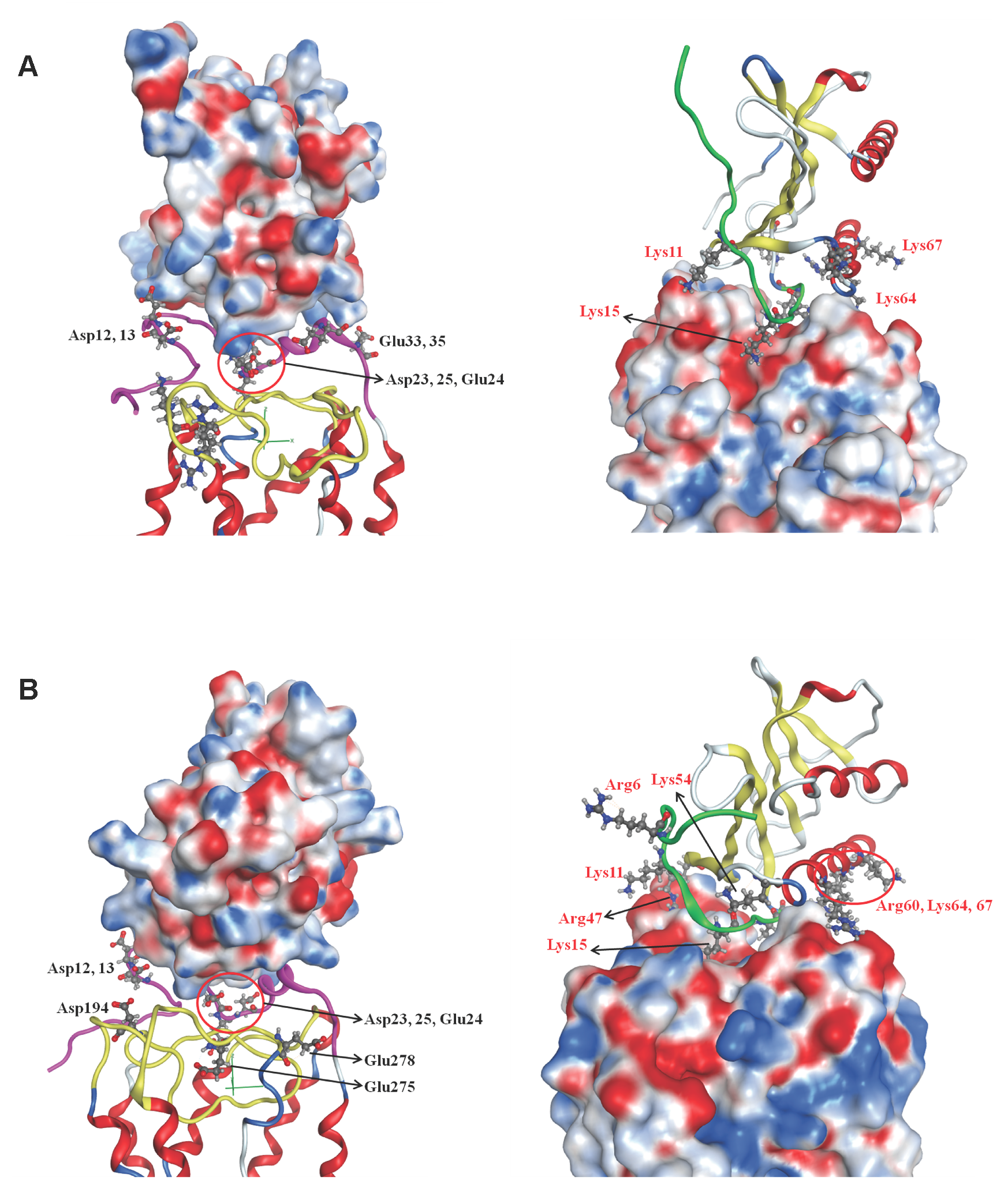

Supplement: Figure S4 — The surface charge distributions of the complex structures. (A) Dimeric CXCL-8 binding with CXCR1 at the initial time. (B) Dimeric CXCL-8 binding with CXCR1 after the 300 ns runs. The complex structure is represented as ribbon structure with the N-loop of the ligand colored green, the N-terminus of receptor colored pink, and the EC-loops colored yellow. Blue color corresponds to positive and red color to negative electrostatic potential. Residues around the binding interface are labeled and shown as sticks, black is for receptor, while red is for ligand. (TIFF) [file pone.0094178.s004.tif]

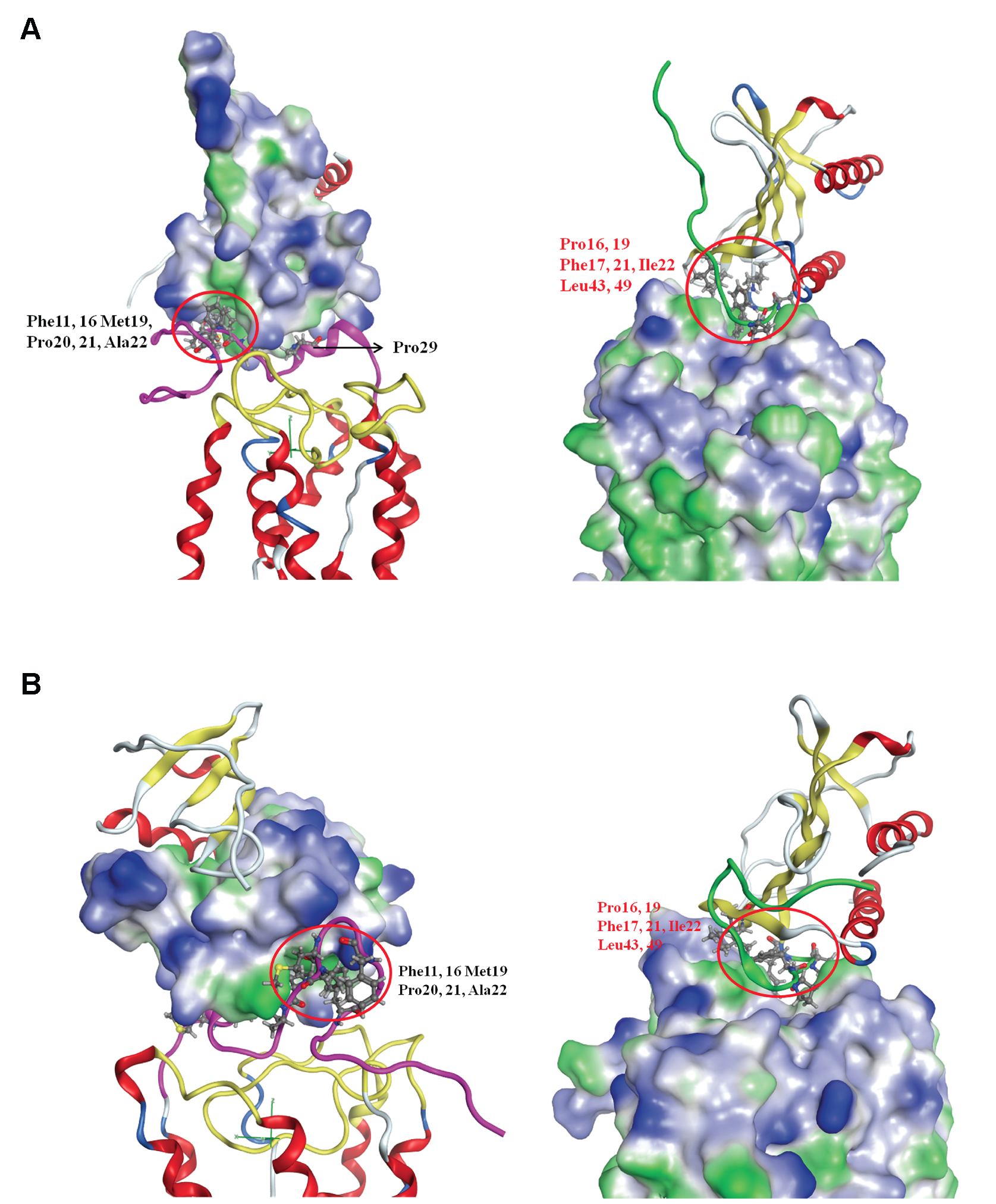

Supplement: Figure S5 — The surface lipophilicity distribution for ligand binding with receptor. (A) Dimeric CXCL-8 binding with CXCR1 at the initial time. (B) Dimeric CXCL-8 binding with CXCR1 after the 300 ns runs. The complex structure is represented as ribbon structure with the N-loop of the ligand colored green, the N-terminus of receptor colored pink, and the EC-loops colored yellow. Blue color represents the hydrophilic part while green color represents hydrophobic part. Residues around the binding interface are labeled and shown as sticks; black font is for receptor, while red font is for ligand. (TIFF) [file pone.0094178.s005.tif]
